# High Utility of Contact Investigation for Latent and Active Tuberculosis Case Detection among the Contacts: A Retrospective Cohort Study in Tbilisi, Georgia, 2010–2011

**DOI:** 10.1371/journal.pone.0111773

**Published:** 2014-11-07

**Authors:** Tsira Chakhaia, Matthew J. Magee, Russell R. Kempker, Medea Gegia, Leila Goginashvili, Ucha Nanava, Henry M. Blumberg

**Affiliations:** 1 University Research Co., LLC. Branch in Georgia, USAID Georgia TB Prevention Project, Tbilisi, Georgia; 2 Division of Epidemiology and Biostatistics, School of Public Health, Georgia State University, Atlanta, Georgia, United States of America; 3 Departments of Epidemiology and Global Health, Rollins School of Public Health, Emory University, Atlanta, Georgia, United States of America; 4 Division of Infectious Diseases, Department of Medicine, School of Medicine, Emory University, Atlanta, Georgia, United States of America; 5 National Center for Tuberculosis and Lung Disease, Tbilisi, Georgia; The Catholic University of the Sacred Heart, Rome, Italy

## Abstract

**Setting:**

The study was conducted at the National Center for Tuberculosis and Lung Diseases (NCTBLD) in Tbilisi, Georgia.

**Objective:**

To assess the utility of contact investigation for tuberculosis (TB) case detection. We also assessed the prevalence and risk factors for active TB disease and latent TB infection (LTBI) among contacts of active pulmonary TB cases.

**Design:**

A retrospective cohort study was conducted among the contacts of active pulmonary TB cases registered in 2010–2011 at the NCTBLD in Tbilisi, Georgia. Contacts of active TB patients were investigated according to an “invitation model”: they were referred to the NCTBLD by the index case; were queried about clinical symptoms suggestive of active TB disease; tuberculin skin testing and chest radiographs were performed. Demographic, laboratory, and clinical data of TB patients and their contacts were abstracted from existing records up to February 2013.

**Results:**

869 contacts of 396 index cases were enrolled in the study; a median of 2 contacts were referred per index case. Among the 869 contacts, 47 (5.4%) were found to have or developed active TB disease: 30 (63.8%) were diagnosed with TB during the baseline period (co-prevalent cases) and 17 (36.2%) developed active TB disease during the follow-up period (mean follow up of 21 months) (incident TB cases). The incidence rate of active TB disease among contacts was 1126.0 per 100 000 person years (95% CI 655.7–1802.0 per 100,000 person-years). Among the 402 contacts who had a tuberculin skin test (TST) performed, 52.7% (95% CI 47.7–57.7%) had LTBI.

**Conclusions:**

A high prevalence of LTBI and active TB disease was found among the contacts of TB cases in Tbilisi, Georgia. Our findings demonstrated that an “invitation” model of contact investigation was an effective method of case detection. Therefore, contact investigation should be scaled up in Georgia.

## Introduction

Investigating the contacts of patients with tuberculosis (TB) is a priority public health measure employed in high-income countries to detect new TB cases and identify those close contacts who would benefit from latent tuberculosis infection (LTBI) treatment to prevent progression to active TB disease [1]. The U.S. Centers for Disease Control and Prevention (CDC) has indicated that contact investigation has played an important role in decreasing the incidence of TB in the U.S. [2]. In contrast, TB contact investigation has typically been a low public health priority in most high TB incidence low- and middle-income countries (LMIC) [3], [4]. In recent years, there has been increasing interest in contact investigation in resource-limited countries. The World Health Organization (WHO) currently recommends contact investigation in two high-risk populations including children <5 years of age and HIV-infected persons, or when the index case has any of the following characteristics: sputum smear positive [SS (+)] pulmonary TB, multidrug-resistant TB (MDR-TB) or extensively drug-resistant TB (XDR-TB), HIV-infection, or is a child <5 years of age [5], [6]. However, WHO indicates that contact investigations may not necessarily be indicated because of competing demands on time and resources and it is frequently not carried out in most LMIC [5], [6].

The country of Georgia has a high burden of TB including MDR-TB disease [7], [8]. In 2012, the incidence rate of TB in Georgia was 116 per 100,000 persons and approximately 9.2% of new cases and nearly a third of retreatment cases had MDR-TB [9]. In 2010–2011, an “invitation” model of contact investigation was implemented: only contacts who presented to health care facilities in conjunction with an index TB case (cases of new or recurrent pulmonary TB in persons from households (HH) or other settings in which others may have been exposed) were investigated [10]. Per National TB Program (NTP) guidelines, contacts did not come in for study follow-up visits nor were visited by health care workers. Currently, most contacts investigated are referred to a health care facility by the TB index case.

In Georgia and most LMICs in the Caucuses region, limited data exists regarding the prevalence of LTBI and the impact of performing contact investigations for TB case finding among HH and close contacts of patients with TB. Previously published epidemiologic investigations of LTBI in Georgia have been conducted only among healthcare workers and internally displaced persons (IDP) [11], [12]. To our knowledge, no data exists regarding the rates of TB among the contacts of active TB patients. Therefore, the overall purpose of our study was to assess the utility of contact investigation for TB case finding. The primary objectives were to 1) determine the prevalence of active TB disease and LTBI at baseline and 2) determine the incidence of active TB disease among contacts of pulmonary TB cases during the follow-up period. A secondary objective of the study was to identify risk factors associated with LTBI and active TB disease among contacts.

## Materials and Methods

### Study design and Setting

A retrospective cohort study was conducted at the ambulatory department of the National Center for Tuberculosis and Lung Diseases (NCTBLD) in Tbilisi, Georgia. Study data for TB patients and their contacts were abstracted from NCTBLD medical records and NTP database from January 2010 through February 2013. Given the retrospective nature of the study, informed consent was not obtained; however, we only used routinely collected programmatic data and all personal identifiers were removed prior to statistical analyses. The Institutional Review Boards of the NCTBLD of Georgia and Emory University reviewed and approved the study protocol.

### Participants

Index cases were defined as cases of new or recurrent pulmonary TB in persons from HH or other setting in which others may have been exposed, as defined by the WHO [5]. Eligible index cases for the study included all patients with pulmonary TB who registered in the ambulatory department of the NCTBLD from January 1, 2010 to December 31, 2011 and who referred contacts to a health care facility for investigation. Index cases with acid-fast bacilli (AFB) SS (+) specimens were instructed by physicians to refer their HH contacts to the NCTBLD. In addition, sputum smear negative [SS (−)] index cases who voluntary referred their contacts to the NCTBLD were also included in this investigation. Contacts were defined as any persons who were exposed to an index case, according to WHO criteria [5]. Eligible contacts for this study included any contacts referred to the NCTBLD by an enrolled index patient including HH (defined as person who shared the same enclosed living space for one or more nights or for frequent or extended periods during the day with the index case during the 3 months before commencement of the current treatment episode [5]) and other non-HH, close contacts (defined as a person who is not in the household but shared an enclosed space, such as a social gathering place, workplace or facility, for extended periods during the day with the index case during the 3 months before commencement of the current treatment episode [5]). All enrolled contacts were investigated during the baseline period – less than 2 months after the index patient TB treatment start date [13].

Contacts were evaluated for clinical symptoms; chest radiography (CXR) and tuberculin skin tests (TSTs) were performed. Nurses at the NCTBLD performed a TST on contact who agreed to be tested using the Mantoux method and the TST was read 48–72 hours after administration [14]. Clinicians used clinical symptoms, the TST result, and chest radiograph findings to identify contacts who were suspected of having active TB disease. All persons suspected of having TB had sputum collected for AFB sputum smear microscopy and culture. All TB screening tests were performed as standard practice within the framework of the NTP and thus there was no charge for the contact. According to the WHO recommendations and per Georgia NTP standards, children aged <5 years who were contacts of drug-susceptible TB index cases and were not diagnosed with active TB disease were offered a 6-month course of isoniazid preventive treatment (IPT) [5].

Within the framework of our retrospective study, prospective follow-up study visits of the contacts were not performed. The data for all contacts during the follow-up period was abstracted from the NTP active TB database. If a contact was registered in the National TB Program as a TB case, this contact was classified as an active TB case. Contacts were classified as TB free at the end of the study period if they were not registered as an active TB case in the NTP database.

### Definitions

The primary study outcomes included the presence of secondary active TB cases and LTBI among contacts. A secondary TB case was defined as active pulmonary or extrapulmonary TB disease among a contact confirmed by a positive AFB culture for *M. tuberculosis* or a clinical diagnosis of TB by a NCTBLD clinician (based on compatible symptoms and radiographic findings). Secondary cases were classified as either co-prevalent or incident TB cases. Co-prevalent TB cases were defined by the presence of active TB disease among contacts during the baseline period. Incident TB cases were defined as the development of active TB disease during the follow-up period among those contacts without active TB disease at baseline. The follow-up period was defined≥2 months after the index patient started therapy for TB up to 2 years or until the end of the study period (February 2013) [13]. For contacts who developed active TB disease, the follow-up period ended on their TB treatment initiation date. Incident TB cases among contacts were confirmed through reviewing the Georgia NTP TB database (NTP surveillance covers nearly all active TB cases in Georgia). A contact with LTBI was defined as any contact person with a positive TST who did not have active TB disease during baseline investigation [5]. An induration of five or more millimeters was defined as a positive TST [14], [15].

Additional data abstracted from the medical record or the Georgian NTP surveillance database included socio-demographic and clinical characteristics of both index cases and contacts. Clinical characteristics included AFB smear status, TB treatment status (newly diagnosed case vs. retreatment TB case) and the presence or absence of MDR TB (defined as resistance to at least isoniazid and rifampin) [16]. All sputum specimens were processed at the Georgian National TB Reference Laboratory in Tbilisi, Georgia according to WHO recommendations [17], [18]. Index patients with higher AFB smear grade were defined as those patients with AFB smear≥2+.

### Statistical methods

Data management and statistical analyses were conducted using SPSS v.19.0 (IBM, USA) and OpenEpi v.2.3.1 (Open source). Separate analyses were performed among co-prevalent TB, incident TB, and LTBI cases. The prevalence of active TB disease among contacts during the baseline period was calculated by dividing the number of co-prevalent TB cases by the number of all contacts in the study. The incidence rate of active TB disease among contacts was calculated by dividing the number of incident TB cases by the total number of person-years of follow-up among all contacts at risk of active TB (i.e., excluding co-prevalent TB cases). The prevalence of LTBI among the contacts during the baseline period was calculated by dividing the number of contacts with LTBI by the total number of contacts who did not have active TB disease and had TST performed during the baseline period. We performed univariate analyses to determine factors associated with active TB disease and LTBI among the contacts. To compare rates of incident active TB among the contacts stratified by index patients' and their contacts characteristics, we used exact methods to calculate unadjusted rate ratios (RR) and 95% confidence intervals (CI) [19]. To assess the factors associated with overall active TB disease as well as with prevalent LTBI among the contacts, Mantel-Haenszel chi-square analysis was used to estimate odds ratios (OR) and 95% CI for all active TB cases and LTBI comparisons. A two-sided p-value <0.05 was considered statistically significant.

## Results

**Figure 1:**
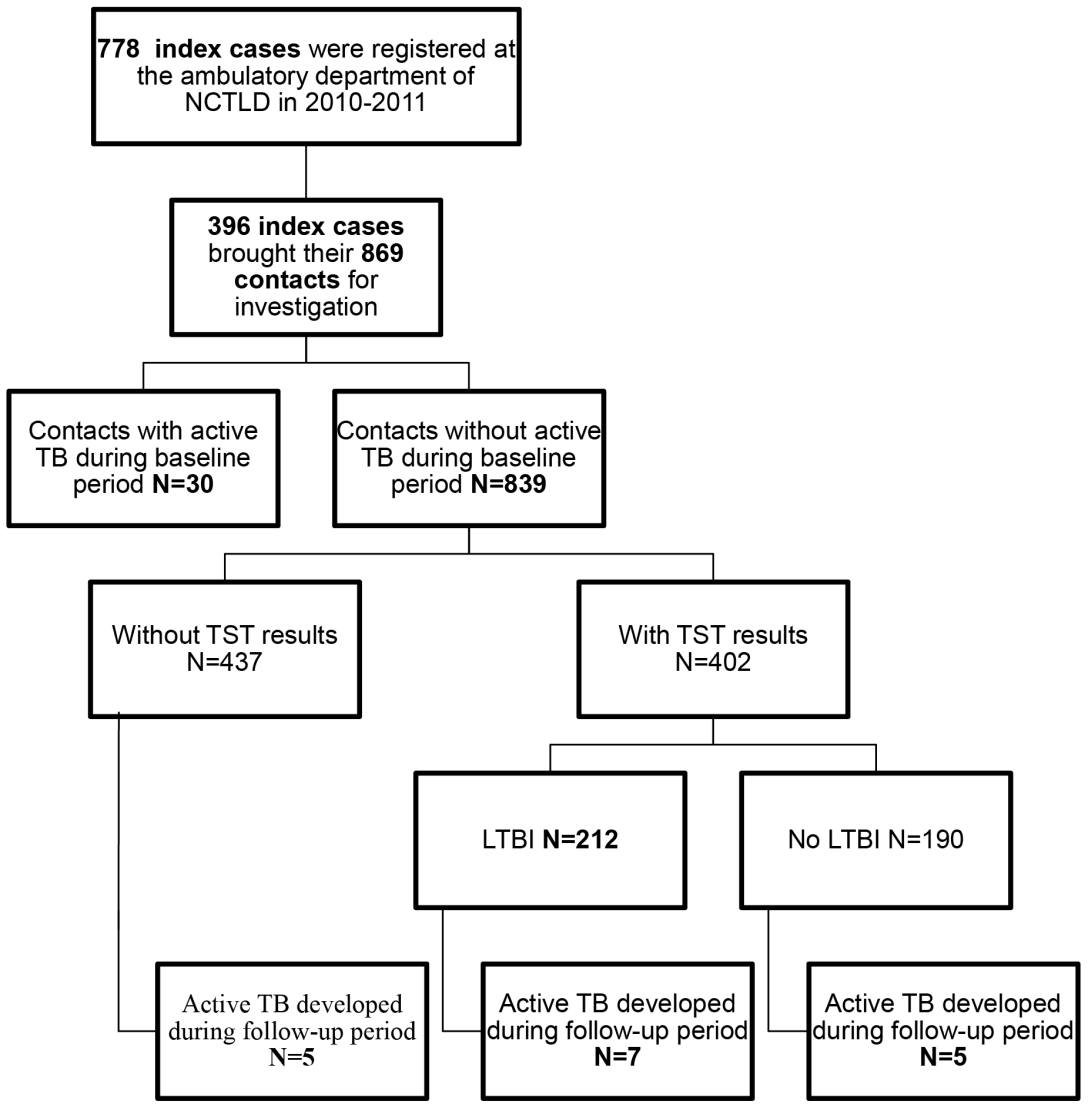
Contact investigation study flow, Tbilisi, Georgia, 2010–2011.

Between January 2010 and December 2011, 778 active pulmonary TB cases were registered at the ambulatory department of the Georgian NCTBLD (Figure 1). Compared to TB patients who did not refer their contacts to a health care facility for investigation (i.e. those who would be eligible as index patients), enrolled index patients were more likely to be AFB SS (+), culture positive, and have MDR TB (Table 1). A total of 396 (50.9%) index TB cases brought in 869 contacts for investigation; a median of 2 (IQR = 2) contacts were referred per index case. The median age of index cases was 34 years (IQR = 20) and 62.6% were male. Most adult index patients were unemployed (87.8%) and 11.4% had a history of incarceration. The large majority of index cases had culture-confirmed TB (95.9%) and positive AFB sputum smear microscopy (89.9%). Two index cases were AFB smear and culture negative. The prevalence of MDR TB among index cases was 27.6% (Table 1). The median age of contacts was 22 years (IQR = 33) including 131 (15.1%) who were <5 years of age (Table 2).

**Table 1:** Socio-demographic and clinical characteristics of index tuberculosis (TB) cases, Tbilisi, Georgia, 2010–2011.

TB Patients' characteristics		TB patients who referred contacts to a health care facility for investigation (index cases)	TB patients who did not referred contacts to a health care facility for investigation	Total	OR (95%CI)
		N = 396 (%)	N = 382 (%)	N = 778	
***All TB patients***					
**Gender**	Male	248 (62.6)	241 (63.1)	489 (62.9)	0.98 (0.73–1.31)
	Female	148 (37.4)	141 (36.9)	289 (37.1)	1
**Age (years)**	0–4	0 (0.0)	0 (0.0)	0 (0.0)	N/A
	5–14	3 (0.8)	7 (1.8)	10 (1.3)	1
	15–24	66 (17.8)	81 (21.2)	147 (19.5)	1.90 (0.47–7.64)
	25–34	122 (32.9)	96 (25.1)	218 (29.0)	2.97 (0.75–11.77)
	35–44	76 (20.5)	87 (22.8)	163 (21.6)	2.04 (0.51–8.16)
	45–54	59 (15.9)	62 (16.2)	121 (16.1)	2.22 (0.55–8.99)
	55–64	29 (7.8)	29 (7.6)	58 (7.7)	2.33 (0.55–9.92)
	> = 65	16 (4.3)	20 (5.2)	36 (4.8)	1.87 (0.42–8.40)
	missing	25	0	25	
**Case definition**	New	281 (78.7)	290 (76.9)	571 (77.8)	1.11 (0.78–1.57)
	Previously treated	76 (21.3)	87 (23.1)	163 (22.2)	1
	Missing	39	5	44	
**AFB smear**	SS (+)	276 (89.9)	146 (39.1)	422 (62.1)	13.84 (9.05–21.16)
	SS (−)	31 (10.1)	227 (60.9)	258 (37.9)	1
	Missing	89	9	98	
**AFB Culture**	Positive	303 (95.9)	224 (66.7)	527 (80.8)	11.65 (6.40–21.23)
	Negative	13 (4.1)	112 (33.3)	125 (19.2)	1
	Missing	80	46	126	
**MDR TB status**	MDR TB	97 (27.6)	55 (14.4)	152 (20.7)	2.26 (1.56–3.27)
	Non MDR TB	255 (72.4)	327 (85.6)	582 (79.3)	1
	Missing	44	0	44	
***Adult index cases*** 1					
**Work status**	Unemployed	281 (87.8)	302 (83.9)	583 (85.7)	1.38 (0.89–2.14)
	Employed	39 (12.2)	58 (16.1)	97 (14.3)	1
	Missing	41	8	49	
**History of incarceration**	Yes	37 (11.4)	39 (11.3)	76 (11.3)	1.01 (0.63–1.63)
	No	288 (88.6)	306 (88.7)	594 (88.7)	1
	Missing	36	23	59	

1 index cases>17 years of age.

**Abbreviations:** AFB–Acid-fast bacilli; SS–sputum smear; MDR–Multidrug-resistant

**Table 2:** Age and gender distribution of the contacts of the index cases with tuberculosis (TB) disease, Tbilisi, Georgia, 2010–2011.

Contacts' Characteristics		Contact of TB patient n = 869	
		*n*	*%*
**Gender**	Male	373	42.9
	Female	496	57.1
**Age (years)**	0–4	131	15.1
	5–14	198	22.8
	15–24	148	17.0
	25–34	112	12.9
	35–44	93	10.7
	45–54	90	10.4
	55–64	57	6.6
	> = 65	40	4.6

### Active TB disease

**Figure 2:**
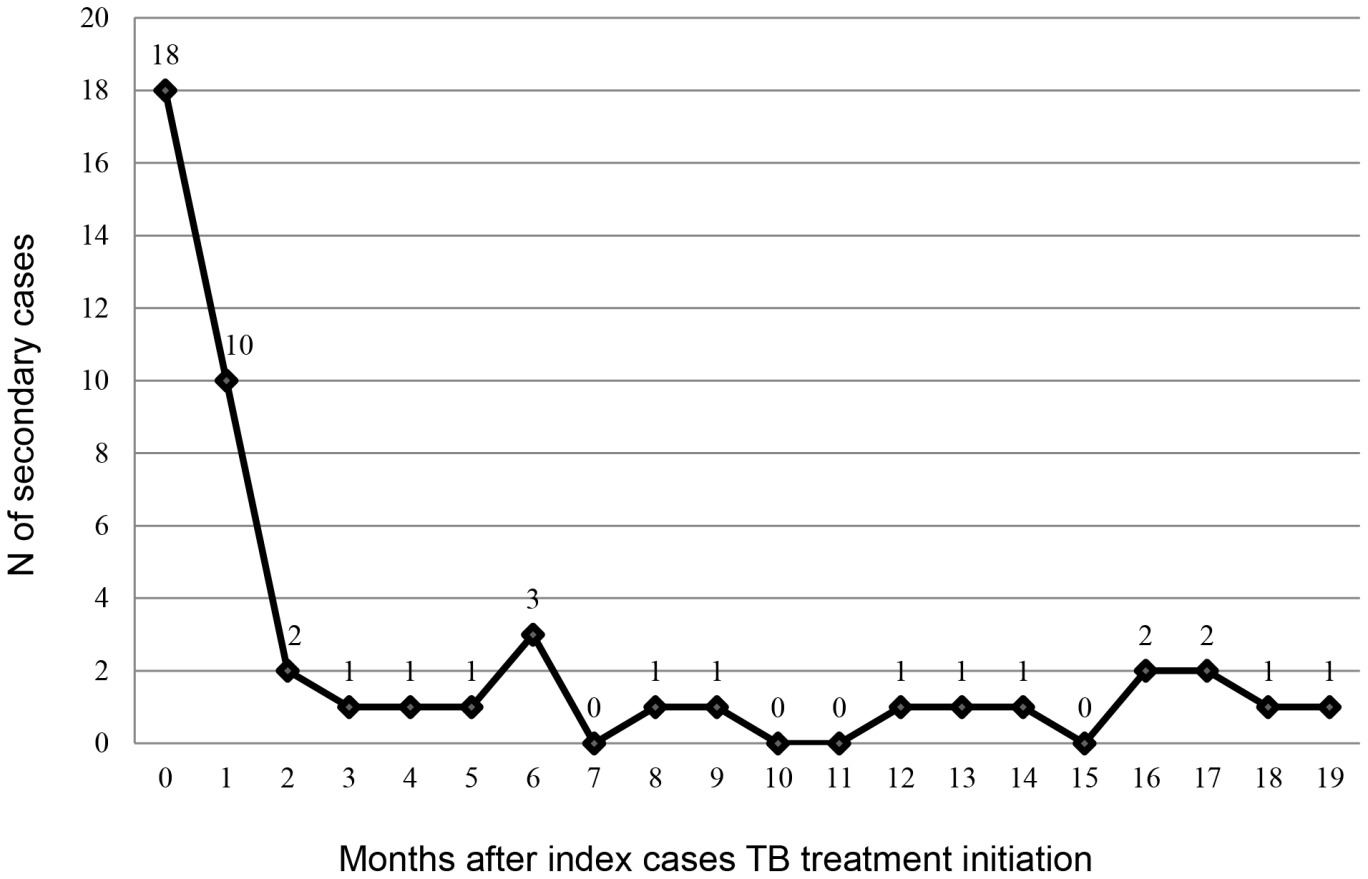
Timing of diagnosis of co-prevalent and incident TB cases among the contacts of index cases, Tbilisi, Georgia, 2010–2011 (n = 47).

Among 869 contacts, 47 (referred by 44 index patients) [5.4% (95% CI 4.0–7.1%)] were found to have active TB at baseline or developed active TB disease during follow-up period. This included 33 cases of pulmonary TB and 14 cases of extrapulmonary TB. Among the 47 contacts with active TB disease, 30 (63.8%)–15 bacteriologically confirmed TB cases and 15 clinically diagnosed TB cases – were found to have active TB during the baseline period (co-prevalent cases) and 17 (36.2%)–8 bacteriologically confirmed TB cases and 9 clinically diagnosed TB cases – developed active TB during the follow-up period (incident TB cases) (Table 3, Figure 1, 2).

**Table 3:** Active TB disease among contacts, Tbilisi, Georgia, 2010–2011.

Months following initiation of TB treatment in index case	Total number of contacts followed, N	Active TB among contacts, N (%)	Person-Years	Rate of active TB disease per 100 000 person years (95%CI)	Rate Ratio (95%CI)
0–2	869	30 (63.8)	N/A	N/A	N/A
3–6	839	6 (12.8)	419.0	1432.0 (525.5–3117.0)	18.40 (2.22–152.80)
7–12	833	3 (6.4)	832.42	360.4 (74.3–1053.0)	4.63 (4.81–44.51)
13–18	830	7 (14.9)	1216.42	575.5 (231.4–1186.0)	7.39 (0.90–60.09)
19–24	623	1 (2.1)	1284.92	77.8 (1.9–433.6)	1

During the follow up period 17 of 869 contacts developed active TB for an overall rate of incident active TB of 1126.0 per 100,000 person years (95% CI 655.7–1802.0). During the follow-up period (mean follow up of 21 months), the rate of developing active TB disease among contacts who did not have TB at baseline was highest 3–6 months after their baseline investigation with a rate of 1432.0 per 100,000 person-years. The rate of developing active TB during this 3–6 month period was significantly higher than during the 19–24 months follow-up period (RR = 18.40, 95% CI 2.22–152.80) (Table 3). Among 129 contacts <5 years old who did not have active TB during the baseline period, 4 developed active TB during follow-up period for a rate of 1806.0 per 100,000 person-years. We did not detect any significant predictors of those at increased risk for developing incident TB disease among contacts (Table 4). Out of 17 contacts with incident TB, 7 contacts had LTBI, 5 contacts did not have LTBI and 5 contacts did not perform TST during baseline investigation (Figure 1). We did not detect any significant difference at increased risk for developing incident TB disease between the contacts with or without LTBI.

**Table 4:** Active incident TB among contacts by age, gender and their index cases' characteristics, Tbilisi, Georgia, 2010–2011.

Socio-demographic and clinical characteristics		N of active incident TB	Rate of active incident TB per 100 000 person years (95%CI)	Rate Ratio (95%CI)
**Contacts' Characteristics**				
**All Contacts**		17	1126.0 (655.7–1802.0)	
**Gender**	Male	7	1070.0 (430.1–2204.0)	1
	Female	10	1169.0 (560.4–2149.0)	1.09 (0.42–2.87)
**Age (years)**	0–4	4	1806.0 (492.1–4624.0)	6.28 (0.70–56.15)
	5–14	1	287.7 (7.3–1603.0)	1
	15–24	6	2317.0 (850.1–5042.0)	8.05 (0.97–66.87)
	25–34	4	2041.0 (556.0–5225.0)	7.09 (0.79–63.46)
	> = 35	2	411.5 (4983.0–1486.0)	1.43 (0.13–5.77)
**LTBI**	Yes	7	1842 (740.8–3796)	1.23 (0.39–3.87)
	No	5	1502 (487.7–3505)	1
**Index cases' characteristics**				
***All Index cases***				
**Gender**	Male	10	1095.0 (525.0–2013.0)	1
	Female	7	1173.0 (471.7–2417.0)	1.07 (0.41–2.82)
**Age (years)**	0–34	11	1533.0 (765.1–2742.0)	2.71 (0.86–8.50)
	≥35	4	566.0 (154.2–1449.0)	1
	Missing	2		
**Case Definition**	Previously treated	4	1499.0 (408.3–3837.0)	1.54 (0.49–4.83)
	New	11	974.4 (486.4–1743.0)	1
	Missing	2		
**AFB smear**	≥SS(2+)	7	1190.0 (478.6–2453.0)	1.74 (0.51–5.94)
	<SS(2+)	4	684.6 (186.5–1753.0)	1
	Missing	6		
**MDR TB Status**	MDR TB	1	289.7 (7.3–1614.0)	1
	non MDR TB	14	1369.0 (748.4–2297.0)	4.73 (0.62–35.93)
	Missing	2		
***Adult index cases*** 1				
**Work status**	Unemployed	13	1182.0 (629.3–2021.0)	1.05 (0.24–4.63)
	Employed	2	1131.0 (137.0–4086.0)	1
	Missing	2		
**History of incarceration**	No	13	1137.0 (605.6–1945.0)	1
	Yes	2	1333.0 (161.5–4817.0)	1.17 (0.26–5.18)
	Missing	2		

1 index cases>17 years of age.

**Abbreviations**: AFB – Acid-fast bacilli; SS – sputum smear; MDR – Multidrug-resistant.

There was an increased risk of co-prevalent and incident active TB disease among the contacts of previously treated index patients (OR 2.15; 95% CI 1.10–4.19) compared to the contacts of newly diagnosed index TB cases. There was a trend towards increased risk of prevalent and incident active TB disease among contacts aged 15–24 years compared to other age groups (RR 2.41; 95% CI 0.92–6.27) but the difference was not statistically significant (Table 5). The co-prevalence of active TB during the baseline period was 3.5% (30/869) and was lower among young children (1.5% in those <5 years old) compared to children and adults (3.8% in those ≥5 years old) (data not shown).

**Table 5:** Active Co-prevalent and incident TB among contacts by age, gender and their index cases' characteristics, Tbilisi, Georgia, 2010–2011.

Socio-demographic and clinical characteristics		Contact Status			OR (95%CI)
		Active TB disease N = 47 (%)	No TB disease N = 822 (%)	Total N = 869 (%)	
**Contacts' Characteristics**					
**All contacts**					
**Gender**	Male	18 (38.3)	355 (43.2)	373 (42.9)	0.82 (0.45–1.49)
	Female	29 (61.7)	467 (56.8)	496 (57.1)	1
**Age (years)**	0–4	6 (12.8)	125 (15.2)	131 (15.1)	1.31 (0.43–3.99)
	5–14	7 (14.9)	191 (23.2)	198 (22.8)	1
	15–24	12 (25.5)	136 (16.5)	148 (17.0)	2.41 (0.92–6.27)
	25–34	7 (14.9)	105 (12.8)	112 (12.9)	1.82 (0.62–5.33)
	≥35	15 (31.9)	265 (32.3)	280 (32.2)	1.54 (0.62–3.86)
**Index cases' characteristics**					
***All Index cases***					
**Gender**	Male	29 (61.7)	496 (60.3)	525 (60.4)	1.06 (0.58–1.94)
	Female	18 (38.3)	326 (39.7)	344 (39.6)	1
**Age (years)**	0–34	26 (59.1)	388 (49.8)	414 (50.3)	1.46 (0.79–2.70)
	≥35	18 (40.9)	391 (50.2)	409 (49.7)	1
	Missing			46	
**Case Definition**	Previously treated	14 (33.3)	144 (18.9)	158 (19.6)	2.15 (1.10–4.19)
	New	28 (66.7)	619 (81.1)	647 (80.4)	1
	Missing			64	
**AFB smear**	≥SS (2+)	18 (56.3)	329 (51.2)	347 (51.5)	1.22 (0.59–2.50)
	<SS(2+)	14 (43.8)	313 (48.8)	327 (48.5)	1
	Missing			195	
**MDR TB Status**	MDR TB	13 (31.0)	192 (25.6)	205 (25.9)	1.30 (0.66–2.55)
	non MDR TB	29 (69.0)	557 (74.4)	586 (74.1)	1
	Missing			78	
***Adult index cases*** 1					
**Work status**	Unemployed	33 (89.2)	603 (86.4)	636 (86.5)	1.30 (0.45–3.75)
	Employed	4 (10.8)	95 (13.6)	99 (13.5)	1
	Missing			68	
**History of incarceration**	No	35 (89.7)	623 (88.1)	658 (88.2)	1.18 (0.41–3.40)
	Yes	4 (10.3)	84 (11.9)	88 (11.8)	1
	Missing			57	

1 index cases>17 years of age.

**Abbreviations**: AFB – Acid-fast bacilli; SS – sputum smear; MDR – Multidrug-resistant.

### Latent TB infection

Among those contacts without active TB during the baseline period, 402 (47.9%) of 839 contacts agreed to have a TST performed and 437 (52.1%) declined to have a TST performed. The prevalence of LTBI among this group that had a TST performed was 52.7% (95% CI 47.7–57.7%) (212 out of 402 had LTBI) (Figure 1). We did not detect any characteristics of index patients or contacts that were statistically significantly associated with risk of LTBI. IPT was taken by 14 (16.9%) of 83 children aged <5 years old who were the contacts of drug-susceptible TB index cases. None of the contacts receiving IPT developed active TB disease, while one child who did not receive IPT developed active TB disease during the follow-up period.

## Discussion

There is increasing interest in the utility of contact investigation in LMIC where contact investigation has traditionally not been part of TB control efforts [20]. In 2010–2011 an “invitation” model of contact investigation (where index patients are asked to invite their contacts to be investigated at a health care facility), was implemented within the NTP of Georgia. Our study demonstrated that an “invitation” model of contact investigation, which is a type of passive contact investigation, was an efficient method. We found a high prevalence of active TB cases (3.5%) among contacts of index patients during the baseline period. The invitation model of contact investigation requires few resources, can be easily implemented into ongoing TB control activities and may significantly increase TB case finding in other LMIC's, which do not currently include contact investigations in their national TB program strategy.

We found the invitation model of contact investigation to be an efficient mechanism to implement TB contact investigations without significantly increasing costs. Nonetheless, previous studies have noted active TB contact investigations to have higher yields. A study performed in rural Malawi reported a higher yield of TB cases that were identified among the HH contacts of SS (+) pulmonary TB cases by using active case finding; TB prevalence by passive case finding among HH contacts was 191 per 100,000 and TB prevalence with active case finding was 1735.0 per 100,000 [21]. Another study from Lima, Peru, concluded that inclusion of active case finding importantly increased the identification of TB cases [22]. Significant more resources are required for active contact tracing which has been a barrier to implementation of contact investigation in many LMIC.

As noted, we found a high prevalence (3.5%) of active TB cases among contacts of index patients (30 TB cases among 869 contacts) during the baseline period. Another 17 contacts developed active TB disease during the follow-up period. TB incidence was similar in contacts with or without LTBI. Random error could account for the non-significance in rate ratios that was observed in our study. Because the sample size of our study population was relatively modest, random error is a possible explanation. Overall, we found a high proportion (5.4%) of active TB cases among contacts of index patients. We also found an increased risk of developing active TB disease among the contacts of previously treated index patients which may be due to very prolonged exposure to an infectious index case. Most of the TB cases among contacts (64%) were co-prevalent TB cases, indicating that active TB disease was identified among contacts during the contact investigation at baseline.

Our study also had important findings regarding LTBI. We observed that LTBI was highly prevalent among contacts of index patients; more than half of the contacts without active TB disease who agreed to undergo TST had prevalent LTBI. The prevalence of LTBI among the general population in Georgia is poorly defined so it is not clear how the prevalence of LTBI in contacts compares to the general population although we hypothesize that it is likely significantly higher among contacts. Despite WHO recommendations that all contacts of non-MDR TB cases who are <5 years of age should receive IPT [5], [23], relatively few children in our study received such treatment for LTBI (15%). The low level of LTBI treatment among young children who were contacts suggests that health care workers in Georgia may benefit from additional training regarding the benefits of IPT and changes should be made to implement current policy and WHO based recommendations on use of IPT in young children. Our findings that IPT was infrequently used for those children <5 years old, who were the contacts of drug-susceptible index cases is also consistent with the knowledge, attitudes, and practices survey conducted among healthcare workers in Georgia. According to this study, many health care workers in Georgia had incomplete knowledge about LTBI [24].

Our findings are the first data reported from Georgia on the utility of contact investigation. The finding of a high prevalence of active TB disease and LTBI among contacts of index TB cases provides data on the utility of contact investigation and in our setting this was done with limited resources using an “invitation” model. A systematic review and meta-analysis of active TB and LTBI among close contacts of index cases with pulmonary TB in LMIC showed that 4.5% of all household contacts had active TB disease at the time of investigation and 51.4% had LTBI [25]. In most reports, incident TB cases among contacts occurred within the first year (particularly within the first 6 months) after detection of the index case [26].

Our study is subject to several limitations. First, because the study included only contacts of index TB cases that received care from NCTBLD in Tbilisi, the cohort may not be representative of the entire country of Georgia. Moreover, contact investigations conducted at the time of the study were passive (“invitation”) and required index patients to refer their contacts to the NCTBLD for evaluation. Consequently, the contacts evaluated in our study may not be generalizable to all TB contacts. However, given the substantial resources needed to conduct investigations on all TB contacts, our “invitation” approach may be a very cost effective way to evaluate contacts and detect new TB cases. Second, because we did not perform genotyping of *M. tuberculosis* strains, we were not able to compare transmission patterns and were unable to definitively determine if transmission occurred from the contacts' index patient or from another source. Future studies that use strain identification of *M. tuberculosis* with genotyping methods among index patients and their contacts are needed to confirm our findings. Third, all TB contacts were not systematically screened for active TB during the follow-up period which could have resulted in an underestimation of the incidence of TB among contacts. Similarly, if contacts developed TB but did not seek care at the NCTBLD the case would not have been identified.

## Conclusion

The burden of active TB disease and LTBI was high among the contacts of active TB cases in this study carried out in Tbilisi, Georgia. We demonstrated that an “invitation” model of contact investigation was an efficient method to expand TB case finding among contacts of index TB cases in a recourse-limited setting. Our results suggest that in LMIC such as Georgia, aggressive and timely contact investigation may efficiently enhance TB case finding among recent contacts. Public health interventions are needed to scale up contact investigations and more aggressively identify contacts of index TB cases.
